# Effect of Filler Particle Size on the Recyclability of Fly Ash Filled HDPE Composites

**DOI:** 10.3390/polym13162836

**Published:** 2021-08-23

**Authors:** Mohammed N. Alghamdi

**Affiliations:** Department of Mechanical Engineering Technology, Yanbu Industrial College, Yanbu Al-Sinaiyah City 41912, Saudi Arabia; alghamdim@rcyci.edu.sa

**Keywords:** fly ash, HDPE, polymer composite, recycling, particle size, mechanical properties

## Abstract

Fly ash polymer composites are innovative high-performance materials that reduce the environmental worries and disposal complications of heavy industry produced fly ash. This study developed and characterized such composites of high-density polyethylene (HDPE) matrices and found that the use of small (50–90 µm) particles of fly ash could give rise to the tensile modulus (~95%) and tensile strength (~7%) of their reinforced composites when compared to neat HDPE materials. While these results themselves convey a strong message of how fly ash can be effectively utilized, this was not the key aim of the current study. The study was extended to examine the effect of fly ash particle size on the recyclability of relevant HDPE composites. The extrusion-based multiple recycling of composites gave slightly lower mechanical properties, primarily due to filler/matrix delamination when large fly ash particles were used. Compared to freshly made fly ash-filled HDPE composites, although using small (50–90 µm) fly ash particles reduced the tensile modulus and tensile strength of recycled composites, the values were still far above those from neat HDPE materials. This novel insight directs the effective utilization of fly ash and provides long-term sustainable and economical solutions for their practical applicability.

## 1. Introduction

Fly ashes are highly abundant synthetic materials produced as byproducts in several heavy industries, including cement [1], petroleum [2], and coal-powered power plants [3,4,5]. Among them, coal-powered power plants produce extremely high quantities of fly ash that are the residue of coal combustion. When coal is totally burnt, the constituents of coal—principally, the oxides of silica and alumina—convert into fly ash. About 80% of ash flies, along with flue gases, become entrapped in bag filters or electrostatic precipitators and are identified as fly ash [6]. The huge quantity of such fly ash has adverse effects on the environment, leading to worse global climate change [7]. For instance, the improper disposal of fly ash to settling ponds may cause environmental contamination, some of heavy metals that are present in fly ash and, if present in high concentrations, are phytotoxic; others are toxic to fish and aquatic organisms [8,9,10]. The environmental contamination due to the leaching of heavy metals from coal fly ash ponds are: phytotoxicity, the contamination of soils and vegetation, and ground and surface water pollutions [6].

To limit the concerns over the global environmental and complex and expensive disposal processes, the utilization of fly ash has become of great importance. Fly ash can be mixed with various materials to make high-value yet inexpensive products. For instance, they can be mixed with concrete for several applications, such as pre-mix, pavements, subbases and dams [11,12]. It has also been used to stabilize road bases and asphalt [13]. There are several advantages when fly ash is mixed with concretes, including a better workability, pump ability, and resistance to alkali-aggregate reaction and a reduction in the heat of hydration, permeability, drying shrinkage, and creep [14]. Fly ash can also limit sulphate attacks and allow good carbonation and corrosion protection while correctly cured [15].

Taking the concept of reinforcing construction materials with fly ash, many researchers have shown that there exists excellent compatibility between fly ash and thermoplastic polymers and developed composites of this kind [16,17,18,19,20]. Other research works have also demonstrated the benefit of using treated fly ash in a wide range of polymer matrices. Such high-performance polymer composites have the potential to be utilized in automotive [21,22] and construction industries [23,24]. Although quite a few innovative strategies are academically published, one key real-world problem associated with those composites that has been overlooked is the recyclability of such composites. Thermoplastic polymer composites have limited lifetimes, and almost 95% of such composites are being landfilled currently [25]. Therefore, it is not only essential to consider non-ecofriendly fly ash to use in polymer composite development but also important to recycle and reuse the composites. Considerations should also be taken in investigating the fabrication or compositional parameters of the composites, namely filler concentration, filler size, and filler/matrix interfacial compatibility.

In this article, the development of fly ash-filled high-density polyethylene (HDPE) composites and the effect of the filler particle size on the thermal, interfacial, and microstructural properties of the composites is initially reported. The study was further guided into a novel investigation that revealed the effect of fly ash particle size on the recyclability of such composites when considering the tensile, thermal, and filler/matrix interfacial properties. This investigation will potentially open a new topic of fly ash-filled polymer composite research as recyclability, which is the key to global circular economy and environmental sustainability.

## 2. Materials and Methods

### 2.1. Materials

Fly ash powders were sourced from Marafiq water and a power plant, Yanbu, Saudi Arabia. Three different size ranges of fly ash (FA) particles were considered: FA1 (50–90 µm), FA2 (90–150 µm), and FA3 (150–250 µm). The high-density polyethylene (HDPE) sample was obtained from Saudi Basic Industries Corporation (SABIC HDPE P6006N), Saudi Arabia.

### 2.2. Experimental Method

**Figure 1:**
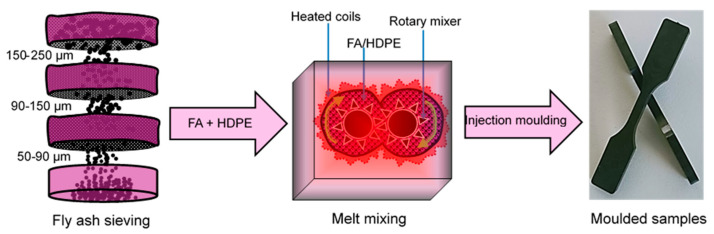
Fabrication method of the FA/HDPE composites with different sized fly ash particles.

As-received fly ash powders were initially sieve-screened from any large particles present. Some (10 wt%) of each FA1, FA2, and FA3 were melt-mixed separately with HDPE by using a HAAKE PolyLab Mixer at 220 °C for 30 min. It was demonstrated in previous works that the use of >10% fly ash further increases the tensile modulus of the FA/HDPE composites but at a cost of reduced tensile strength [26,27]. Both of these mechanical properties are important considerations; therefore, the current study uses 10% fly ash, which is considered the optimum in terms of having a high tensile modulus and tensile strength. Once the composite mixture was taken out and cooled at room temperature, they were named Composite 1 (contains 10% FA1), Composite 2 (contains 10% FA2), and Composite 3 (contains 10% FA3). All the fly ash-mixed HDPE (FA/HDPE) composite samples, along with the pure HDPE, were then injection-moulded (HAAKE MiniJet II) at 240 °C for 10 sec to form dumbbell-shaped samples for tensile testing. Four units of each FA/HDPE composite type (and neat HDPE) were prepared and tested to evaluate the reproducibility of the tensile properties. Figure 1 shows a schematic flow diagram of the sample fabrication method. Similar samples were also used to perform the thermogravimetric analysis (TGA) and differential scanning calorimetry (DSC) tests. The fracture surface of the samples were also studied by cryogenically (at −196 °C) breaking them with liquid nitrogen. A scanning electron microscope (SEM) was used to evaluate the extent of the interfacial adhesion of the fly ash particles and HDPE matrix.

### 2.3. Recycling of Composites

The freshly made samples were then recycled by using an extrusion plastometer (Thermo Haake MeltFlixer LT Germany). The recycling was repeated four times when the composite samples, after each recycling, were stored for characterization. The DSC, SEM, and tensile tests were performed on all the recycled samples to evaluate their corresponding properties that were obtained from freshly made samples.

## 3. Results and Discussion

### 3.1. Thermal Degradation and Crystallinity

**Figure 2:**
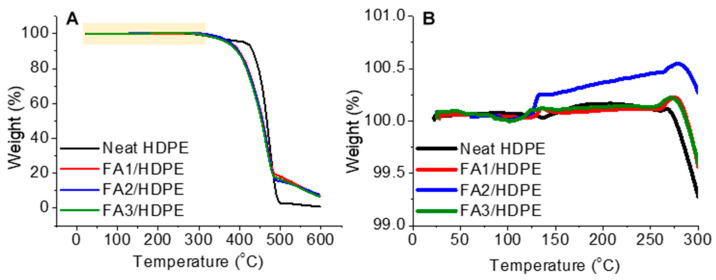
Thermogravimetric degradation of freshly made neat HDPE and FA/HDPE composite samples: (**A**) overall degradation pattern and (**B**) understanding the sample degradation pattern at the temperature near to the melt-processing temperature of the samples of interest.

Thermogravimetric tests were performed only for freshly made neat HDPE and FA/HDPE samples to understand their degradation behaviours. The samples were heated from 25 °C to 600 °C at a heating rate of 5 °C/min (Figure 2A). The tests were conducted in the air so that process can replicate the melt processing-related degradation of the polymer. The neat HDPE sample started to lose weight around 275 °C (as can be seen in Figure 2B) and fully degraded at around 510 °C (Figure 2A). In contrast, the polymer portion of the FA/HDPE samples was ~90% thermally degraded at 510 °C, leaving the fly ash particles undamaged. A further increase in the temperature caused the thermal degradation of the fly ash particles too but not a complete loss of the weight. The key reason for such behaviour is the high thermal stability of the metal particles within the fly ash component. However, the primary objective of these thermogravimetric tests was to investigate the composite degradation behaviour at or near the melt processing temperature. Figure 2B shows the enlarged view of TGA curves at a temperature region that just shows the starting temperature of thermal degradation. An insignificant weight increase in the initial phase of the degradation curve was observed, which might be due to the buoyancy effect of the TGA equipment. This buoyancy effect provided an upward force on the composite samples, produced by the surrounding air environment. During this process, the density of the atmosphere of the TGA chamber decreased with the increasing temperature, resulting in a sample weight gain [28]. However, since the melt processing temperature used in this study was within the range of 220–240 °C, a much closer look was paid at such temperature regions on the TGA curve and found that the extent of the thermal degradation was negligible for all sample types. Therefore, the used melt processing temperature was anticipated to be safe for producing high-value polymer composites.

**Figure 3:**
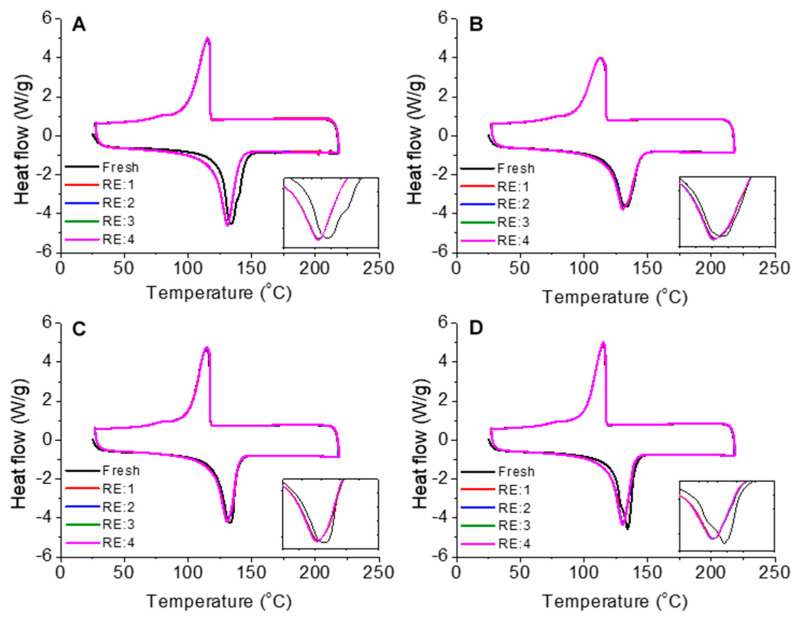
DSC thermograms (5 consecutive heating/cooling cycles): (**A**) Neat HDPE, (**B**) FA1/HDPE, (**C**) FA2/HDPE, and (**D**) FA3/HDPE.

Once the fresh HDPE and FA/HDPE samples were fabricated and thermogravimetrically tested, the samples were subjected to recycling by using an extrusion plastometer, without any pre-treatment. Four repetitive recycling cycles (namely RE: 1, RE: 2, RE: 3, and RE: 4) were performed at 240 °C while using a 5-kg compressive load (ISO 1133). Filament-shaped materials were dispensed and cooled down to room temperature. It is obvious that the remelting will break the crystal alignment of the polymer chains within the HDPE matrix; therefore, DSC tests were performed to evaluate the extent of the crystallinity of the recycled materials (Figure 3). In all cases, the fresh samples provided slightly higher melting points compared to the remelted ones, depicting the loss of crystal contents within the HDPE matrices. Further remelting the samples seldom provided different melting peaks, which agreed that these materials could be recycled multiple times without significantly reducing the polymer crystallinities. Table 1 shows the core results evaluated from the DSC tests, composing the deviations obtained from different melting cycles. The degree of crystallinity was estimated by considering the heat of fusion of a perfect polyethylene crystal (293 J/g) [29]. The extent of composite crystallinities was much preserved for all the recycling steps, particularly having the least deviation for recycling the FA1/HDPE composite samples. The uniformity of particle distribution might also provide a fairly consistent crystal orientation throughout the volume of composite samples. The concept of composite recyclability was taken from the crystallinity studies prior to attempting the recycling of the composites of current interest and investigating their mechanical and microstructural properties.

### 3.2. Mechanical Properties

**Figure 4:**
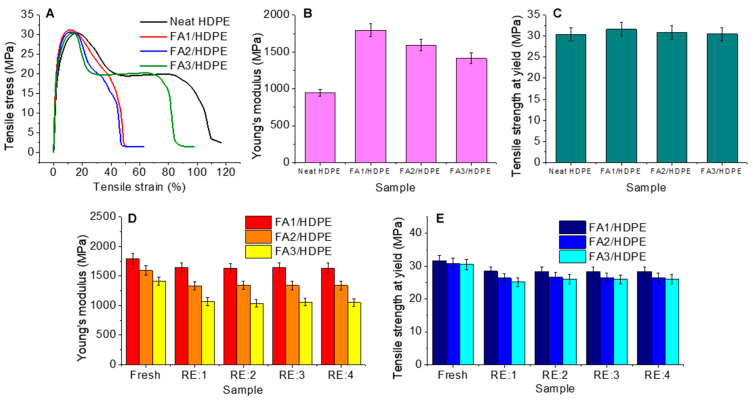
Tensile test results: (**A**) stress-strain curves of freshly made injection moulded samples, (**B**) Young’s modulus of fresh samples, (**C**) tensile strength of fresh samples, (**D**) comparative Young’s moduli of fresh and recycled composites, and (**E**) comparative tensile strength of the fresh and recycled composites. (Error bars show the deviation of tensile results obtained from four different samples).

Initially, fresh injection-moulded neat HDPE and FA/HDPE samples were undergone for tensile testing. As can be seen in Figure 4A–C, Young’s modulus of neat HDPE samples was found to be ~910 MPa. Reinforcing FA1 within the HDPE matrix gave a rise of Young’s modulus to ~1775 MPa, providing a ~95% increase, which is much higher than the previously reported results of similar composites [26,30,31,32]. Using FA2 and FA3, which have larger particle sizes, gave a comparatively lower modulus rise to the FA/HDPE composites. For instance, compared to the neat HDPE sample, the Young’s modulus of the FA2/HDPE and FA3/HDPE composites were found to be ~68% and ~49% higher, respectively. The phenomenon might be attributed to the fact that fly ash particles were much uniformly distributed within the HDPE matrix, without no or a lesser formation of agglomerated particles clusters. Agglomerated fillers are one of the key reasons behind the low mechanical properties of relevant polymer composites. This hypothesis can also be verified while investigating the results of the tensile strength at the yield (Figure 4C). Uniform dispersion of the fillers also helped to enhance the FA/HDPE tensile strength when compared to the neat HDPE polymer. These results also indicated that the composites are of high filler/matrix interfacial adhesion, limiting the chance of premature material failure under tension.

While confirming the high-performance nature of FA/HDPE composites, further investigation was carried out to understand the effect of recycling on the tensile properties of the composites of current interest. The extrusion-based recycling of composites gave an idea of material degradation while undergoing a remelting process, aligning to the principle of the results obtained from the DSC tests. Figure 4D,E shows the comparative Young’s modulus and tensile strength of the fresh and recycled composites. FA1/HDPE composites are most suitable for recycling in terms of retaining the highest extent of tensile properties compared to the FA2/HDPE and FA3/HDPE composites. Taking the DSC results into account, a loss of crystallinity contributes similarly to all three composites, particularly in the first recycling attempt. From the tensile test results, first-time recycling of the FA1/HDPE composite gave a sharp decrease in the Young’s modulus (~10%) and tensile strength (~8%). Compared to the FA1/HDPE composite, the FA2/HDPE and FA3/HDPE composites provided a much higher decrease in Young’s modulus (~19% and 32%, respectively) and tensile strength (~17% and 21%, respectively), contradicting the loss of crystallinity found from the DSC results. The reason for a higher decrease in the tensile properties might be the loss of filler/matrix interfacial adhesion due to the bigger fly ash particles that have a lower surface area, as demonstrated in previous research works [33,34]. Further recycling the samples provided highly persistent tensile properties even after four consecutive recycling periods. These results further validate the effectiveness of smaller fly ash particles in terms of recycling the reinforced polymer composites. However, a further microstructural analysis is needed to validate the claim.

### 3.3. Filler/Matrix Morphology

**Figure 5:**
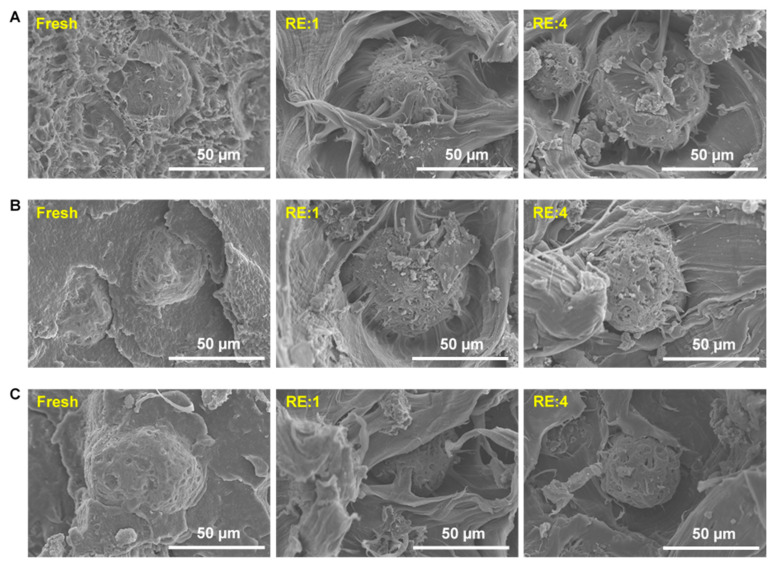
SEM images of cryogenically fractured fresh and recycled composites: (**A**) FA1/HDPE, (**B**) FA2/HDPE, and (**C**) FA3/HDPE.

Microstructural SEM imaging of the FA/HDPE composites was performed using the cryofracture surfaces of such composites (Figure 5). Cryogenic fracturing was used to avoid shear or tension-assisted manipulation of the filler/matrix interfaces. The fractured surface of the samples was sputter-coated with platinum prior to the microscopic observation. The high-vacuum SEM mode was used while applying a 10-KeV electron beam on the fractured surface. The key aim of the SEM imaging was to evaluate the qualitative information of the filler–matrix interfacial compatibility that usually dominates the mechanical properties of the composites.

As seen from the fresh composites, the filler/matrix interfacial adhesion is quite high, and the fly ash particles were well-impregnated within the HDPE matrices even after cryogenic fracturing. First-time recycling (RE: 1) negatively changes the extent of interfacial adhesion, resulting in a higher portion of fly ash particles delaminated from the surrounding matrix. However, the interface delamination is lowest in the FA1/HDPE composite after the first recycling period (Figure 4A), finely validating the results obtained from the tensile tests. It is worth noting from the SEM images that similar-sized fly ash particles were focused on maintaining the consistency of the filler/matrix adhesion. Further recycling the composites (SEM images shown for the fourth-time recycled composites, RE: 4) barely changed the filler/matrix interfacial adhesion, validating the consistent tensile results obtained from RE: 1–4. In most of the melt-processed thermoplastic composites, the filler/matrix interfacial shear strength primarily contributed to the overall mechanical properties of the composites [35,36].

## 4. Conclusions

In this paper, fly ash-filled HDPE composites were developed, and their physical, mechanical, and microstructural properties were investigated. The initial results showed that the use of small (50–90 µm) fly ash particles gave a rise of the Young’s modulus (~95%) and tensile strength (~7%) of their reinforced composites when compared to neat HDPE materials. The filler/matrix interface imaging of such a composite shows a high adhesion between them that validated the tensile test results. However, the aim of the current study was to investigate the feasibility of the melt recyclability of the composites and the effect of filler size on the mechanical properties of the recycled composites. The extrusion-based recycling of the composites gave slightly lower mechanical properties, mostly owing to the filler/matrix delamination when large fly ash particles were used. Micro- or nanoparticles are vulnerable in forming agglomerated clusters that could lead to the premature failure of their reinforced composites. Compared to freshly made FA/HDPE composites, using small (50–90 µm) fly ash particles reduced the Young’s modulus and tensile strength of the recycled composites; however, the values were still far above the ones from neat HDPE materials. Most importantly, the effective recyclability was valid for four consecutive cycles, giving a strong insight into the technical feasibility of such composite systems. Therefore, it can be concluded that the use of small fly ash particles (~50–90 µm, used in this study) is able to provide high-performance FA/HDPE composites, along with the potentiality of recycling them into good value materials.

Although the key of this study lies in the potential recyclability of FA/HDPE composites, there is still much to discover in terms of the fundamental chemical properties of the filler/matrix interface. One possible method is to conduct Fourier-transform infrared (FTIR) spectroscopy to evaluate the micro-interfacial properties, and it is anticipated to demonstrate such analysis results in our future publication. It is also anticipated to conduct an X-ray diffraction (XRD) analysis of the fresh and recycled composites to compare their crystallographic structures, further linking them to the mechanical properties of the relevant composites. Considering the trend of the tensile properties observed from the currently investigated FA/HDPE composites, it would also be worthwhile to consider smaller fly ash particles (<50 µm) for making better-performing composites.

## Figures and Tables

**Table 1:** DSC analysis results of the FA/HDPE composites.

Sample	Fresh/Recycling Steps	Melting Temperature (°C)	Area of Melting Peak (J/g)	Degree of Crystallinity (%)
Neat HDPE	Fresh	133.97	169.6	57.8
	RE: 1–4	131.2 (±0.5)	166.3 (±0.8)	56.7 (±0.7)
FA1/HDPE	Fresh	133.19	159.1	54.3
	RE: 1–4	130.91 (±0.8)	153.3 (±0.6)	52.3 (±0.5)
FA2/HDPE	Fresh	133.16	158.6	54.1
	RE: 1–4	130.79 (±0.3)	153.0 (±0.7)	52.2 (±0.2)
FA3/HDPE	Fresh	134.11	158.2	53.9
	RE: 1–4	130.41 (±0.6)	151.9 (±0.5)	51.8 (±0.4)

## Data Availability

The data presented in this study are available on request from the author.
